# COVID-19 pandemic waves: how prepared is West Africa for managing a high COVID-19 caseload? Urgent actions needed

**DOI:** 10.11604/pamj.2021.40.249.31107

**Published:** 2021-12-21

**Authors:** Virgil Kuassi Lokossou, Chukwuma David Umeokonkwo, Stanley Okolo, Patrick Mboya Nguku, Nanlop Ogbureke, Issiaka Sombie

**Affiliations:** 1 ECOWAS Regional Center for Disease Surveillance and Control, Nigeria,; 2 Department of Community Medicine, Alex Ekwueme Federal University Teaching Hospital, Abakaliki, Ebonyi State, Nigeria,; 3 African Field Epidemiology Network, Abuja Nigeria,; 4 West African Health Organization, Bobo-Dioulasso, Burkina Faso

**Keywords:** COVID-19, waves, vaccination, genomic surveillance

## Abstract

The ECOWAS Region and the world have learnt a lot in the last year and a half concerning the pandemic. As the pandemic continues to evolve, the region needs to put together all these lessons in other to better protect its people, rebuild its economy and strengthen the regional health security for better regional prosperity. We reviewed the response mounted by the region from January 2020 to July 2021 and the existing body of knowledge. We recommend that the region quickly increase the COVID-19 immunization coverage, sustain the enhance genomic surveillance, improve testing and the strengthen point of entry surveillance.

## Commentary

COVID-19 has caused since 2020 an unprecedented regional crisis, including thousands of lives lost, public health systems in shock and economic and social disruptions. As of July 2021, ECOWAS Region reported over 532,258 cases and over 7,004 deaths with increasing notification of new variants capable of worsening the epidemiologic trends.

ECOWAS Region has focused its response to COVID-19 pandemic on the implementation of the Test-Trace-Isolate-Treat strategy supported by important measures in Infection and Prevention and Control (IPC) and logistics. This involved expanding testing capacity, digitizing contact tracing, reinforcing point of entry surveillance, rolling out equitable vaccination, enhancing treatment and care particularly for the most vulnerable to improve the national capacity to fight devastating COVID-19 waves [1].

In this commentary, we are looking at urgent actions that ECOWAS Countries can take to mitigate the impact of COVID-19 crisis on lives and livelihoods in West Africa. COVID-19 can be a very challenging situation with the occurrence of major COVID-19 crisis in many places around the world, West Africa needs to urgently learn from COVID-19 crisis that happened in Brazil, Peru and India in order to prepare adequately for a possible high surge of new COVID-19 cases [2-4]. Managing COVID-19 pandemic in this context require governments take decisive, quick and critical actions to predict, assess, prepare to respond to future waves of the pandemic. As West Africa anticipates future waves, member states need to adapt science-guided mitigation measures to curb the spread of SARS-CoV-2.

This opinion piece aimed to provide some guidance to orient ECOWAS Countries. We undertook an extensive review of literature including regional strategic meeting reports, guidelines, and recommendations on ongoing and effective public health response interventions during COVID-19 crisis in ECOWAS Region. We focused our reflections around critical response pillars that is of high important during the Management of COVID-19 crisis in West African context.

### Vaccinations

Vaccines are crucial to the containment of the COVID-19 pandemic; however, they work synergistically with scientifically proven effective interventions like use of face mask, proper IPC practices and active surveillance of infection and evolution of different variants of the virus. Sourcing COVID-19 vaccine has been a great challenge in Africa in general as over 99% of its vaccine need is sourced from outside the continent and there is marked disparities in COVID-19 vaccine rollout in Africa at the regional level. Several factors have been adduced for the disparity which were not limited to challenges with pre-purchase agreements with manufacturers and stockpiling of doses to the high income country (HIC) population, exports restrictions placed on vaccines, vaccine raw materials, and on delivery supplies such as syringes and glass bottles [5].

In West Africa, Member States have sourced their vaccines through the COVAX provision, African Vaccines Acquisition Task Team (AVATT) Initiative and through bilateral arrangements. The proportions procured as at end of June 2021 is still a far cry to what is needed to vaccinate at least 70% of the population in the region. Most member states have still focused their vaccination efforts at the high-risk group, comprising those aged 45 years and older and healthcare workers. This should remain the immediate short-term goal since younger age groups have been more affected and it may probably save the most lives and keep the health service delivery ongoing without systemic failures.

Member states should accelerate vaccination effort to ensure available vaccines are quickly and efficiently made available to the populace with minimal waste. Vaccination effort should remain staggered from high risk to low risk and should be coupled with massive advocacy and risk communication to minimize vaccine hesitancy. Member states need to increase a risk communication effort to engage community and all the actors to accept vaccinations. Improved vaccination coverage will have immediate benefit for the health of the populace and improve faster economic recovery. Several challenges including vaccines hesitancy, institutional mistrust, corruption, fragile rumours management systems, insufficient funding for operations and campaigns are limiting vaccines rolling out in the region [6, 7].

There is an urgent need to operationalize the ECOWAS Vaccines Exchange Platform and the ECOWAS Vaccines Revolving fund for pooled procurement both for vaccines and other essential vaccination supplies from different sources proposed by the Authority of the ECOWAS Head of States in January 2021 and for improving vaccine uptakes. This will create opportunity for pooling financial resources both from member states, partners and cooperating organizations in West Africa. It will also provide a better bargaining strength than individual countries, in addition to help in creating regional resilience in case things get out of hands.

### Surveillance/Containment Strategies

**Figure 1:**
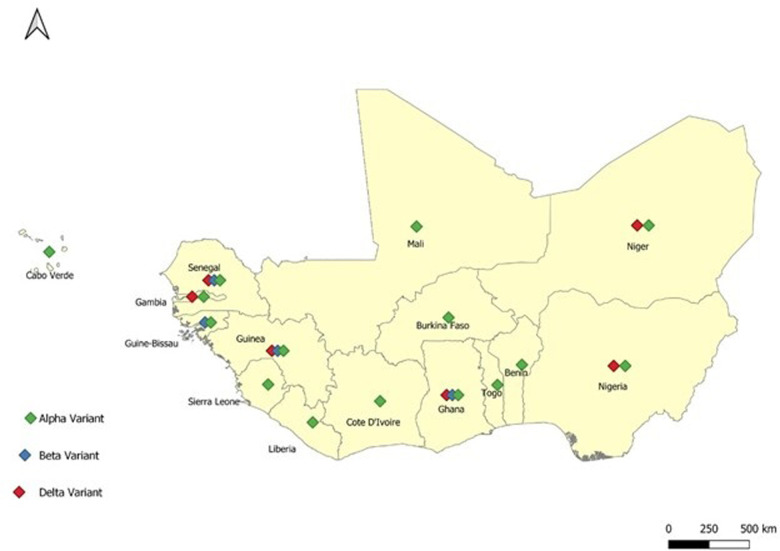
the distribution of the different variants of COVID-19 in the ECOWAS Region as at July 2021

**Figure 2:**
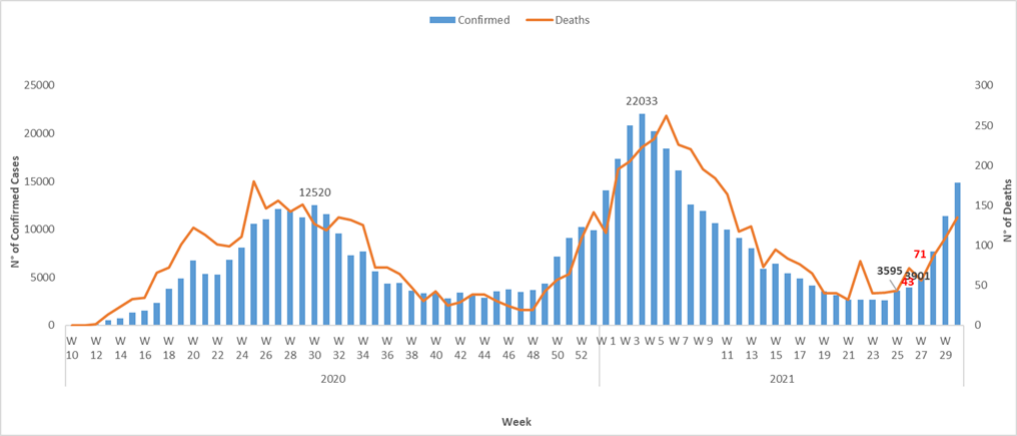
the weekly evolution of COVID-19 cases in West Africa

As part of ongoing surveillance, member states aspire to improve testing capacity, strengthen point of entry surveillance and broaden containment measures [1]. Travellers from nations which are currently experiencing third wave with new variants of concerns should be placed on enhanced surveillance to limit the introduction and spread of these variants in the region. There is an urgent need to foster the operationalization of national and regional enhanced genomic surveillance of all the variants of the virus in the region according to the existing and agreed protocols between the World Health Organization, Africa Centers for Disease Control and Prevention and the West African Health Organization. This is particularly important as more member states report the presence of beta and delta variants (Figure 1) which may be responsible for the raising number of cases (Figure 2). In addition, there is a need to enhance community level surveillance for respiratory diseases through the community health workers.

**Coordination:** it remains a core component of incident management structure during the Management of COVID-19 pandemic. ECOWAS Countries should build on existing structures and platforms within the region and at national levels. Member States established ad hoc coordination mechanisms both at political, strategic, and technical level to efficiently combat COVID-19 pandemic including the use of Public Health Emergency Operation Centers.

There is need to improve coordination at subnational and community levels especially in high transmission area towards a “Whole of Society” response [8]. COVID-19 pandemic waves can rapidly evolve and reach the level of a humanitarian crisis. ECOWAS Member States should be focusing their efforts on coordinating an international humanitarian response to improve health system capacity through expanding cooperation with NGOs, UN Organization already present in the region, accepting regional and international cooperation and humanitarian support, and utilizing access to IMF and World Bank funding mechanisms while leveraging on already existing national assets. There is a need to keep an eye on training more public health experts capable of managing COVID-19 crisis. A surge pool of experts in all the arm of incident management structures should be assembled and ready for quick mobilization as the need arises.

**Laboratory and testing:** the region has over the last one year increased the number of laboratories with capacity to test for COVID-19 from 2 to more than 500. Moving further, ECOWAS Member States need to focus on validating and deploying rapid diagnostic tests especially in border areas and ramp up testing capacity. Improved testing has helped better understanding of the pandemic and drivers of cases spikes and assess the effectiveness of the response. There should be improved capacity to genomic studies by adopting and implementing agreed protocols to monitor the different variants of the virus in the region. As at 11^th^ of May 2021 six different variants has been already identified in the different countries in the region (Figure 1). The region should continue to refine the testing strategy to meet the national and regional circumstances. There is a need to quickly validate the existing rapid diagnostic test kits for COVID-19 and deploy them where appropriate.

**Case management:** managing the rapid rise in severe COVID-19 cases is one of the critical challenges that face any country because of the demands associated with such a complex situation. The direct and indirect effects on health infrastructure are usually enormous. Case management in African context are facing numerous challenges including low capacity of intensive care unit, low oxygen production and delivery, few and poorly equipped isolation and treatments centres especially at subnational level and lack of adequate human resources and medical commodities [9].

Member states are encouraged to make deliberate effort in stockpiling essential commodities, scaling up oxygen production and supply capacities, build up capacities and mobilize adequate and skilled workforce including Community Health Workers and be ready to respond promptly and decisively in order to limit the impact on human life´s if such situation arises. Governments, regional and global agencies should support provision of oxygen generators and concentrators, similarly that vaccines are being scaled up through global partnerships like COVAX and AVATT. National Health authorities should update clinical treatment protocols based on available evidence, improve quality of care in Isolation Treatment centres and promote innovative approaches like the use of E-Health.

**Risk communication and community engagement (RCCE):** this remains the gold standard for preventing infections during COVID-19 crisis even after the availability of the vaccines. Unfortunately, human behaviour due to a low perception of risk has contributed to low uptake of preventive measures and fuelled the spread of COVID-19 in communities., one major challenge in ECOWAS Region is to convince communities that there is a problem and that they need to take personal responsibilities to limit the spread of the pandemic [10]. Other challenges also included the inadequate levels of public trust on response strategies and authorities and the lack of conducive environment, as well as the inadequate public health policies to promote uptake of public health measures. This is where crafting the right messages and community engagement becomes indispensable. In addition, lessons and recommendations from previous crisis should be used to respond to high surge of cases, including the rapid involvement of community influencers and the implementation of Social and Behaviour Change Communication (SBCC) programmes tailored to local context. Finally, Risk Communication and Community Engagement (RCCE) approaches should be evolved based on the transmission dynamics at the community level [10].

Non-pharmaceutical interventions (NPIs) include all measures or actions, outside of vaccines and medicines, that can implement to prevent, slow the spread of COVID-19 in Community. Whilst focusing and expanding the health service that will save lives during crisis, the biggest impact will come from NPIs. ECOWAS Member Community should foster the implementation of mitigation strategies, include personal, environmental and community NPIs. Nationwide or subnational lockdowns are neither sustainable nor effective in West Africa. Though temporary application of these measures can be effective in preventing and controlling hotspots by reducing human interactions.

**Conclusion:** COVID-19 is a long-game; the best time to start implementing effective preparedness and response measures to address the COVID-19 crisis in West Africa may have been several years ago, but the next best time is now. We need sustained political commitment to implement the available effective interventions (Annex 1) at global, regional and national level [6, 11, 12]. ECOWAS Countries have limited capacity for critical care buttressing the need to focus on containment strategies and enhanced surveillance. Improving coordinated strategies between countries, enhancing vaccination of high-risk categories, containing the introduction of new virulent strains and scaling up availability of critical essential health commodities and supplies are encouraged to minimize the risk of occurrence of important wave and its impact in West Africa.

## Annex

**Annex 1**: summary of key interventions recommended to ECOWAS Member states for preparing to respond COVID-19 response [11]
